# Influence of exercise on quantity and deformability of immune cells in multiple sclerosis

**DOI:** 10.3389/fneur.2023.1148106

**Published:** 2023-05-18

**Authors:** Undine Proschmann, Puya Shalchi-Amirkhiz, Pauline Andres, Rocco Haase, Hernán Inojosa, Tjalf Ziemssen, Katja Akgün

**Affiliations:** Multiple Sclerosis Center, Center of Clinical Neuroscience, Department of Neurology, University Hospital Carl Gustav Carus, Dresden University of Technology, Dresden, Germany

**Keywords:** exercise, multiple sclerosis, lymphocytes, leukocytosis, cell deformability, real-time-deformability cytometry, disease modifying treatment

## Abstract

**Objective:**

The study aimed to investigate the effect of exercise on immune cell count and cell mechanical properties in people with multiple sclerosis (pwMS) on different disease-modifying treatments (DMT) vs. healthy controls (HCs).

**Methods:**

A cohort of 16 HCs and 45 pwMS, including patients with lymphopenia (alemtuzumab and fingolimod) as well as increased lymphocyte counts (natalizumab), was evaluated for exercise-mediated effects on immune cell counts and lymphocyte deformability. As exercise paradigms, climbing stairs at normal speed or as fast as possible and cycling were used, while blood samples were collected before, immediately, and 20 as well as 60 min post-exercise. Immune cell subtypes and lymphocyte deformability were analyzed using multicolor flow cytometry and real-time deformability cytometry.

**Results:**

An increase in lymphocytes and selected subsets was observed following exercise in HCs and all pwMS on different DMTs. Patients with lymphopenia exhibited an increase in absolute lymphocyte counts and immune cell subsets till just below or into the reference range. An increase above the upper limit of the reference range was detected in patients on natalizumab. Exercise-induced alterations were observable even in low and more pronounced in high-intensity physical activities. Lymphocyte deformability was found to be only mildly affected by the investigated exercise regimes.

**Conclusion:**

People with multiple sclerosis (PwMS) treated with alemtuzumab, fingolimod, and natalizumab respond to acute exercise with a comparable temporal pattern characterized by the increase of immune cell subsets as HCs. The magnitude of response is influenced by exercise intensity. Exercise-mediated effects should be considered when interpreting laboratory values in patients on immunomodulatory therapy. The impact of exercise on biophysical properties should be further elucidated.

## 1. Introduction

Multiple sclerosis (MS) is an autoimmune disease characterized by a dysbalance of adaptive immune cells. Over the last three decades, there has been a rapid expansion of therapeutic agents for MS. Many of these therapeutic options target lymphocyte count and function which may be linked to immunosuppression. Frequent examination of blood cell counts has become important to monitor treatment effects and treatment-limiting factors (1, 2). In addition, the characterization of immune cell phenotype and functional analysis of immune cells have gained importance in many immune-mediated diseases, particularly in a personalized therapeutic approach (3, 4).

Physical exercise is known to increase the circulating leukocyte count by mobilizing the marginated pool and should be considered when interpreting laboratory findings (5–8). The amplitude of the immune response to acute short exercise depends on the duration and intensity of the physical workload (9–11). Both innate and adaptive immunity are altered by physical activity (12, 13). Exercise immunology in MS is gaining increasing interest and some exercise-derived immunomodulatory effects have been already postulated including alterations of the cytokine profile and Toll-like receptor expression (14–17). Even if it is assumable that physical activity leads to an acute immune response in pwMS during disease-modifying therapy (DMT), there are no studies that have discussed this question in this specific group of patients and its relevance in the interpretation of lab results until now.

Alongside immune cell frequencies, biophysical properties of cells and tissues are increasingly used to identify and understand pathological mechanisms in several diseases and immunomodulatory treatments (18, 19). For example, cell stiffness was previously found to be sensitive to malignant transformation in tumors (20). Cell deformability serves as a potential innovative biomarker for cell function, especially as new technologies have been evaluated to enable a reliable and valid measurement (21). The exercise-induced catecholamine release may impact the cytoskeleton of immune cells and modify the adhesion of these cells to endothelium leading to lymphocyte demargination (22–24). Hitherto, knowledge about the impact of distinct exercise levels on cell deformability is limited.

Alemtuzumab (ATZ, Lemtrada^®^, Sanofi Genzyme, Diegem, Belgien), fingolimod (FTY, Gilenya^®^, Novartis Pharma AG, Basel, Switzerland), and natalizumab (NAT, Tysabri^®^, Biogen, Cambridge Massachusetts, USA) are approved treatments for pwMS. The mode of action of these DMT including pan-lymphocyte depletion and interference with lymphocyte trafficking highlights the need for frequent blood examinations and determination of the immune status (25–28).

In this study, we aimed to characterize the response pattern of immune cells and lymphocyte deformability to different exercise regimes in pwMS treated with ATZ, FTY, and NAT in comparison to HCs.

## 2. Materials and methods

### 2.1. Subjects

A total of 16 HCs and 45 pwMS treated with either ATZ (*n* = 15), FTY (*n* = 15), or NAT (*n* = 15) were included. HCs and pwMS treated with ATZ, FTY, and NAT were instructed either to climb 72 stairs (four flours) at normal speed, to climb 72 stairs as fast as possible or to cycle an ergometer for 20 min with weight-adapted resistance (1,2 Watt per kilogram bodyweight). Patient characteristics are presented in Table 1.

**Table 1:** Characteristics of healthy controls (*n* = 16) and people with multiple sclerosis (*n* = 45).

Climbing stairs normal							
	Age years, mean (SD)	Gender female/male	EDSS mean (SD)	Disease duration years, mean (SD)	Therapy duration months, mean (SD)	Time since last ATZ infusion months, mean (SD)	Number of ATZ cycles mean, (SD)
HC	26.0 (3.6)	3/3	NA	NA	NA	NA	NA
ATZ	30.6 (6.0)	3/2	2.2 (0.8)	3.0 (1.8)	30.3 (33.7)	11.0 (7.1)	2.2 (1.1)
FTY	26.4 (3.0)	2/3	2.0 (0.6)	3.0 (1.2)	17.6 (12.2)	NA	NA
NAT	26.4 (5.0)	5/0	1.3 (0.4)	6.2 (3.1)	58.6 (16.3)	NA	NA
Climbing stairs fast							
HC	23.1 (1.2)	1/5	NA	NA	NA	NA	NA
ATZ	29.4 (2.9)	4/1	1.8 (0.3)	9.0 (4.5)	6.2 (5.8)	3.8 (1.8)	1.2 (0.4)
FTY	27.3 (3.1)	2/3	1.8 (0.5)	6.5 (4.0)	34.2 (31.4)	NA	NA
NAT	28.2 (3.0)	3/2	1.4 (0.2)	5.4 (3.3)	30.8 (27.6)	NA	NA
Cycling							
HC	23.8 (2.4)	3/2	NA	NA	NA	NA	NA
ATZ	29.4 (6.6)	2/3	1.8 (0.5)	3.6 (2.9)	7.8 (5.6)	5.2 (2.3)	1.2 (0.4)
FTY	31.2 (6.1)	3/2	1.5 (0)	10.2 (5.5)	40.2 (17.9)	NA	NA
NAT	22.6 (2.8)	3/2	2.0 (0.9)	5.6 (2.4)	61.2 (37.6)	NA	NA

ATZ, alemtuzumab; EDSS, expanded disability status scale; FTY, Fingolimod; HC, healthy control; NA, not applicable; NAT, natalizumab; pwMS, people with multiple sclerosis.

### 2.2. Sample collection

Venous blood was drawn from each subgroup of HCs and pwMS with a 21-gauge butterfly needle into a sodium citrate and ethylenediaminetetraacetic (EDTA) K blood tube (Sarstedt, Nümbrecht, Germany) at four time points including prior to exercise, immediately, and 20 as well as 60 min post-exercise.

### 2.3. Ethical approval

The study was performed according to the Declaration of Helsinki and approved by the Ethics Committee of the Faculty of Medicine of the Dresden University of Technology, Germany. All participants provided written informed consent.

### 2.4. Fluorescence-activated cell sorting

Whole fresh blood samples were prepared immediately after collection for immunophenotyping. Immune cell populations were characterized by surface staining with the following fluorescence-labeled antibodies according to the manufacturers´ instructions: HLADR-BV510 (G46-6; BD Bioscience), CD3 APC Cy7 (SK7, BD Bioscience), CD45-FITC (HI30, BD Bioscience), CD14-PECF 594 (MφP9, BD Bioscience), CD19-PE Cy5 (HIB 19, BD Bioscience), CD8-PerCP CY5.5 (RPA-T8, BD Bioscience), CD4-PE Cy7 (RPA-T4, BD Bioscience), and CD56-BV785 (5.1H11, Biolegend). For the lysis of red blood cells, FACS lysing solution (BD Bioscience) was added. Viability dye (VD, eBioscience) staining was used to evaluate apoptotic cells. Cell frequencies were evaluated on the LSR Fortessa cytometer (BD Bioscience, San Jose, CA, USA). Gating strategy for cells is presented in Supplementary Figure S1.

### 2.5. Real-time deformability cytometry

Whole fresh blood samples were analyzed by real-time deformability cytometry (RT-DC) approximately 30 min after blood withdrawal. RT-DC was performed as described previously (21). Briefly, 50 μL of citrate anti-coagulated blood were diluted in 950 μL measurement buffer (MB) Carrier B (Zellmechanik Dresden, Dresden, Germany) and mixed gently by manual rotation of the sample tube. Cells in the MB were then taken up into a 1 mL syringe, placed on a syringe pump (neMESYS, Cetoni, Korbussen, Germany), and connected with the sample inlet of the microfluid chip. For measurements, a total flow rate of 0.08 L/s with a sample flow rate of 0.02 μL/s and a sheath flow rate of 0.06 μL/s was used. Images of the cells in the channel were acquired in a region of interest of 250 × 80 pixels at a frame rate of 2,500 fps using a pulsed LED for image exposure and a high-speed complementary metal-oxide semiconductor camera. In total, we analyzed a minimum of 3,500 and up to 1,00,000 cells per sample. The ShapeOut software (Zellmechanik Dresden, Dresden, Germany) was used for analysis. Only cells with a deviation of the actual cell area of less than 5% from the area tracked by the algorithm were included.

### 2.6. Statistical analysis

The normal distribution of data was visually assessed using quantile–quantile plots and confirmed by the Shapiro–Wilk test. Quantitative population characteristics were presented as measures of central tendency (mean, median), followed by the standard deviation (SD) or range.

Longitudinal patient data were analyzed separately for each exercise intensity level by the generalized linear mixed model (GLMM) with gamma distribution and log link function because of the right-skewed distribution pattern of the data and timepoint, DMT/HC, and interaction of timepoint and DMT/HC as fixed effects of the model. The GLMM was also applied for comparison between exercise groups. A *p*-value of <0.05 was considered to be statistically significant. For pairwise comparisons, contrast tests with Fisher’s least significant difference procedure were applied. Statistical analyses were performed using the IBM SPSS software for MAC (version 25.0, IBM Corporation, Armonk, NY) and GraphPad Prism (version 7; GraphPad Software, La Jolla, California).

## 3. Results

### 3.1. Exercise-induced alterations in leukocyte and lymphocyte subsets

**Figure 1:**
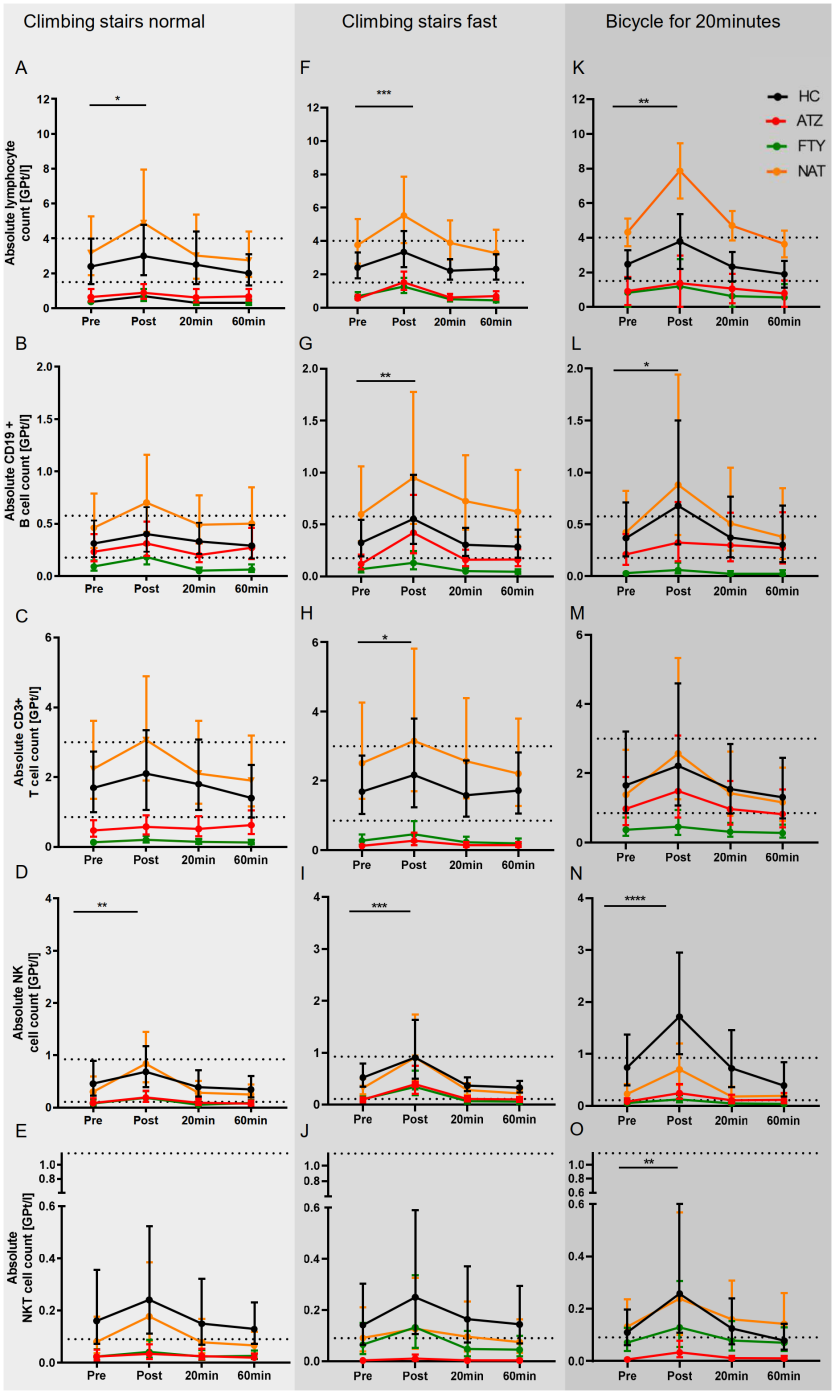
Immune response to distinct exercise levels in HCs and pwMS. The mean of absolute cell concentrations with 95% CI are depicted for HCs (labeled black), pwMS under treatment with ATZ (labeled red), FTY (labeled green), and NAT (labeled orange) for distinct exercise levels including low intensive exercise **(A–E)**, short-term high intensive exercise **(F–J)** and endurance exercise **(K–O)**. Data points of measurements were at rest before (pre), immediately (post), and 20 and 60 min after exercise. The reference range is delimited by dashed lines. Data were analyzed by generalized linear mixed models for repeated measures. Asterisks indicate the statistically significant difference between the time points pre-and post-exercise (*p < 0.05, **p < 0.01, ***p < 0.001).

Based on the mechanism of action, lymphocyte count and its subsets were significantly lower in the ATZ and FTY group compared to HC and even below the reference range, whereas the NAT group presented higher counts compared to HC. All of the investigated cell populations, including lymphocytes, CD19+ B cells, CD3+ T cells, CD4+ T cells, CD8+ T cells, activated CD3+ cells, NK cells, NKT cells, monocytes, and granulocytes, peaked immediately post-exercise and then returned to baseline levels within 1 h post-exercise in all subgroups. The absolute lymphocyte (*p* = 0.026) and NK cell count (*p* = 0.001) were found to be the only cell types with a significant increase immediately after climbing stairs at normal speed as a main effect in overall groups (Figures 1A–E).

The temporal pattern of leukocytosis upon climbing stairs as fast as possible was found to be similar to the first exercise level. However, significant changes were observed in more cell subsets, including absolute lymphocyte (*p* < 0.001), CD19+ B cell (*p* = 0.003), CD3+ T cell (*p* = 0.038), and NK cell (*p* < 0.001) count as main effect overall groups (Figures 1F–J).

The temporal pattern of leukocytosis upon cycling was comparable with the two other exercise regimes. A significant increase in absolute cell count was documented for lymphocytes (*p* = 0.001), NKT cells (*p* < 0.01), and NK cells (*p* < 0.0001) as the main effect overall groups (Figures 1K–O).

In an additional analysis, we compared the immune cell counts between the distinct exercise levels over the entire observation period. Pre-exercise mean immune cell counts were similar between the exercise regimes. Over the entire observation period, the mean absolute lymphocyte count was significantly higher upon cycling than climbing stairs normally (*p* < 0.001) or fast (*p* < 0.05). Absolute CD8+ T cell count was significantly higher upon climbing stairs fast compared to normal (*p* < 0.01). Absolute monocyte cell counts were significantly higher upon climbing stairs fast (*p* = 0.001) and cycling (p < 0.05) compared to climbing stairs normally.

### 3.2. Shift to normal lymphocyte counts in patients on FTY and ATZ post-exercise

An increase in lymphocyte counts and their subpopulations could be documented immediately post-exercise in HCs and pwMS. An exercise-induced shift of lymphocyte counts toward the lower limit of reference range up to normal levels was observed in patients with medication-associated lymphopenia. It is of particular interest that such alteration was already elicited by low-intensity exercise.

### 3.3. Lymphocyte counts above the upper limit of the reference range in patients on NAT

An increase in lymphocytes and subpopulations was seen in patients on NAT too. However, patients with lymphocyte counts within the reference range exhibited an increase in lymphocyte levels even above the upper limit of the reference range immediately post-exercise. This response pattern was seen after all exercise levels.

### 3.4. Lymphocyte deformability

**Figure 2:**
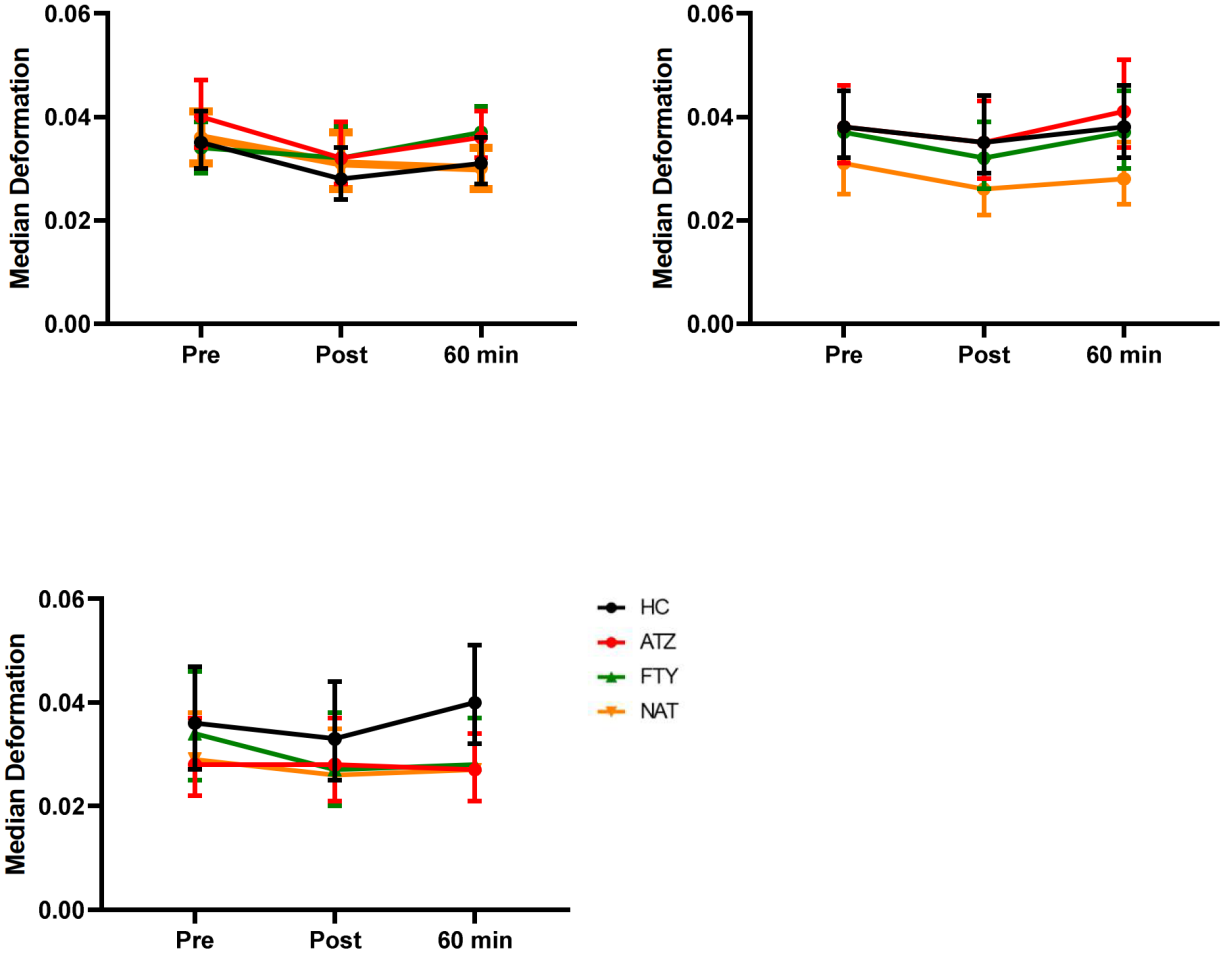
Effect of distinct exercise levels on lymphocyte deformability. Mean lymphocyte deformability with 95% CI is depicted for HC (labeled black), pwMS treated with ATZ (labeled red), FTY (labeled green), and NAT (labeled orange) for climbing stairs slowly **(A)**, climbing stairs fast as possible **(B)** and cycling for 20 min **(C)**. Data points of measurements were at rest before and immediately post-exercise as well as 60 min after exercise. Data were analyzed by generalized linear mixed models for repeated measures.

Lymphocyte deformability assessed immediately after exercise demonstrated no significant changes in all exercise regimes (Figure 2).

## 4. Discussion

The aim of the present study was to investigate the immune response pattern and magnitude to different exercise modes in HCs and pwMS at treatment with ATZ, FTY, and NAT. For the evaluation of alterations in lymphocyte stiffness, the novel RT-DC technique that allows quantitative fingerprinting of cells based on their physical properties was used.

Immunomodulating therapies as state-of-the-art treatments for various autoimmune disorders such as MS require frequent monitoring of the immune status and immunophenotyping (29). Although exercise-induced leukocytosis is a well-established phenomenon and exercise immunology in pwMS is gaining increasing interest with the first randomized controlled trial investigating the effects of exercise in pwMS, data regarding the immune response in pwMS, particularly during DMT, are limited until now (5, 30, 31). Thereby, it is important to characterize exercise-induced leukocytosis in pwMS especially during lymphocytopenia and leukocytosis associated DMT to identify possible falsely high lymphocyte counts. While treatment with ATZ and FTY is associated with treatment-induced lymphopenia, NAT is linked to leukocytosis. ATZ as a humanized monoclonal antibody (mAb) selectively depletes circulating CD52-expressing lymphocytes followed by a distinctive pattern of lymphocyte repopulation with a rebalanced immune system (4, 25, 32). FTY acts as a sphingosine-1-receptor modulator that prevents lymphocyte egress from lymph nodes impairing peripheral lymphocyte recirculation (26, 33–36). FTY results in a reduction of absolute lymphocyte count including specific T cell subsets (36–40). The monoclonal antibody NAT binds the α4-subunit of the α4β1-integrin on leukocytes, thus preventing transmigration throughout the blood–brain barrier into the central nervous system (27). All major lymphocyte subpopulations were found to be significantly increased in peripheral blood due to NAT treatment (41, 42).

We aimed to gain knowledge about the immunologic response of pwMS to different exercise regimes focusing on absolute immune cell counts relevant for lab monitoring of DMT in daily practice. We could demonstrate that pwMS and HCs respond to the exercise to a comparable extent with increased immune cell counts immediately after exercise and a return to baseline levels within 1 h. Additionally, we found that the exercise-induced changes in pwMS were similar during different DMTs including therapies associated with lymphocytopenia. For pwMS treated with FTY and ATZ, we observed an exercise-associated shift of the lymphocytopenia level as defined by the National Cancer Institute Common Terminology Criteria for Adverse Event or even an increase up to normal lymphocyte counts for all exercise levels. Patients on NAT exhibited an exercise-induced increase of lymphocytes up to levels of the upper limit of the reference range.

Consistent with previous observations, we revealed that cells of the innate immune system are preferentially elevated in circulation while cells of the adaptive immune system show a much slower magnitude of response (10). While significant changes were limited to lymphocytes and NK cells in the low-level exercise regime, cycling for 20 min resulted in significant absolute cell count increases of lymphocytes, NKT, and NK cells. Climbing stairs as fast as possible was linked to the most significant changes in cell counts including lymphocytes, CD19+ B cells, CD3+ T cells, and NK cells. An additional analysis comparing the three exercise regimes with each other revealed significantly higher lymphocyte, CD8+ T cell, and monocyte counts over the total observation period in the more intense than in the low-level exercise regime. Although these findings are limited by a small sample size, they are in line with previous findings which suggested an influence of exercise intensity on the magnitude of response (10). The difference between short-lasting high-intensity exercise and longer, less extensive exercise may be caused by stronger adrenergic activation in the brief high extensive exercise. NK cells as part of the innate immune system are known to be the leukocyte subset with the largest increase and NK as well as NKT cells have been previously identified as highly responsive to catecholamine-induced mobilization (43). Similar to these findings, NK cells and NKT cells were found to represent the highest degree of change in our study cohort with more pronounced increases in the higher intense exercise regimes. We could demonstrate that leukocyte subsets are differentially mobilized into the peripheral blood in both HCs and pwMS treated with different DMT.

Cell morphology and mechanics are known to be inherent markers of cell function (44). Mechanical properties of leukocytes play an important role in margination within blood vessels (45, 46). The alteration of lymphocyte stiffness for leukocyte trafficking might be mediated by the immune system (47). More and more studies illuminate the relevance of disease-related alterations of cellular deformability and observed that cells become less deformable in patients suffering from different disease conditions (18–20, 48). Distinct stiffness profiles have been already revealed for malignant cells and their healthy counterparts in solid tumors and leukemias (18, 20, 48). COVID-19 disease was linked with increased lymphocyte stiffness which could contribute to the high incidence of vascular occlusion and pulmonary embolism (49). Additionally, the determination of cellular biophysiological properties was found to be a potential parameter to distinguish breast cancer subpopulations in their metastatic preference suggesting a correlation between stiffness and organotropism (50). However, knowledge about exercise-induced alterations in lymphocyte deformability is scarce. Here, we found no statistically significant change in the mechanical properties of lymphocytes in the evaluated groups for the different exercise intensity levels. Considering the postulated effects of catecholamines and glucocorticoids on the mechanical properties of leukocytes being linked with cell softening, more pronounced effects of exercise on mechanical properties have been expected (47). Further studies are needed in order to recapitulate the effect of exercise on cell deformability.

In conclusion, the immune system was found to show plasticity in response to exercise in both HCs and pwMS with a comparable temporal response pattern. The magnitude of response seems to depend on the exercise workload. Although our data are limited by a small sample size, our findings indicate that regular blood draws during DMT should be performed after resting for a minimum of 20 min in order to avoid invalid blood values, in particular during DMT associated with lymphocytopenia. The impact of exercise on mechanical cellular properties needs to be further elucidated.

## Data availability statement

The raw data supporting the conclusions of this article will be made available by the authors, without undue reservation.

## Ethics statement

The studies involving human participants were reviewed and approved by Ethics committee of the Faculty of Medicine of the Dresden University of Technology, Germany. The patients/participants provided their written informed consent to participate in this study.

## Author contributions

KA and TZ: study concept and design. UP and PA: acquisition of data. UP and PS-A: analysis and interpretation of data. UP, KA, and TZ: drafting of the manuscript. PS-A, PA, RH, and HI: critical revision of the manuscript for important intellectual content. UP and RH: statistical analysis. KA and TZ: study supervision. All authors contributed to the article and approved the submitted version.

## Funding

UP was supported by the Else Kröner Forschungskolleg Dresden. The Article Processing Charge (APC) was funded by the joint publication funds of the TU Dresden, including Carl Gustav Carus Faculty of Medicine, and the SLUB Dresden as well as the Open Access Publication Funding of the DFG.

## Conflict of interest

UP received speaker fee from Merck, Biogen and Bayer and personal compensation from Biogen and Roche for consulting service. RH has received travel compensation from Celgene and Sanofi. HI received speaker fee from Roche. TZ reports consulting or serving on speaker bureaus for Biogen, Celgene, Roche, Novartis, Celgene Merck and Sanofi as well as research support from Biogen, Novartis, Merck and Sanofi. KA reports consulting or serving on speaker bureaus for Roche, Sanofi, Merck, Alexion, Teva, Biogen, BMS and Celgene for consulting service.

The remaining authors declare that the research was conducted in the absence of any commercial or financial relationships that could be construed as a potential conflict of interest.

## Publisher’s note

All claims expressed in this article are solely those of the authors and do not necessarily represent those of their affiliated organizations, or those of the publisher, the editors and the reviewers. Any product that may be evaluated in this article, or claim that may be made by its manufacturer, is not guaranteed or endorsed by the publisher.

## Abbreviations

ATZ: alemtuzumab
DMT: disease-modifying treatment
EDTA: ethylenediaminetetraacetic
EDSS: expanded disability status scale
FACS: fluorescence-activated cell sorting
FTY: fingolimod
GLMM: generalized linear mixed model
HC: healthy control
MB: measurement buffer
min: minutes
MS: multiple sclerosis
NAT: natalizumab
NK: natural killer
pwMS: people with multiple sclerosis
RT-DC: real-time deformability cytometry
s: seconds
SD: standard deviation
Th: T helper
