# Supplementary figures and images for: Influence of exercise on quantity and deformability of immune cells in multiple sclerosis
Source: Front Neurol. 2023 May 18;14:1148106. doi: 10.3389/fneur.2023.1148106 (PMC10232764; doi:10.3389/fneur.2023.1148106)

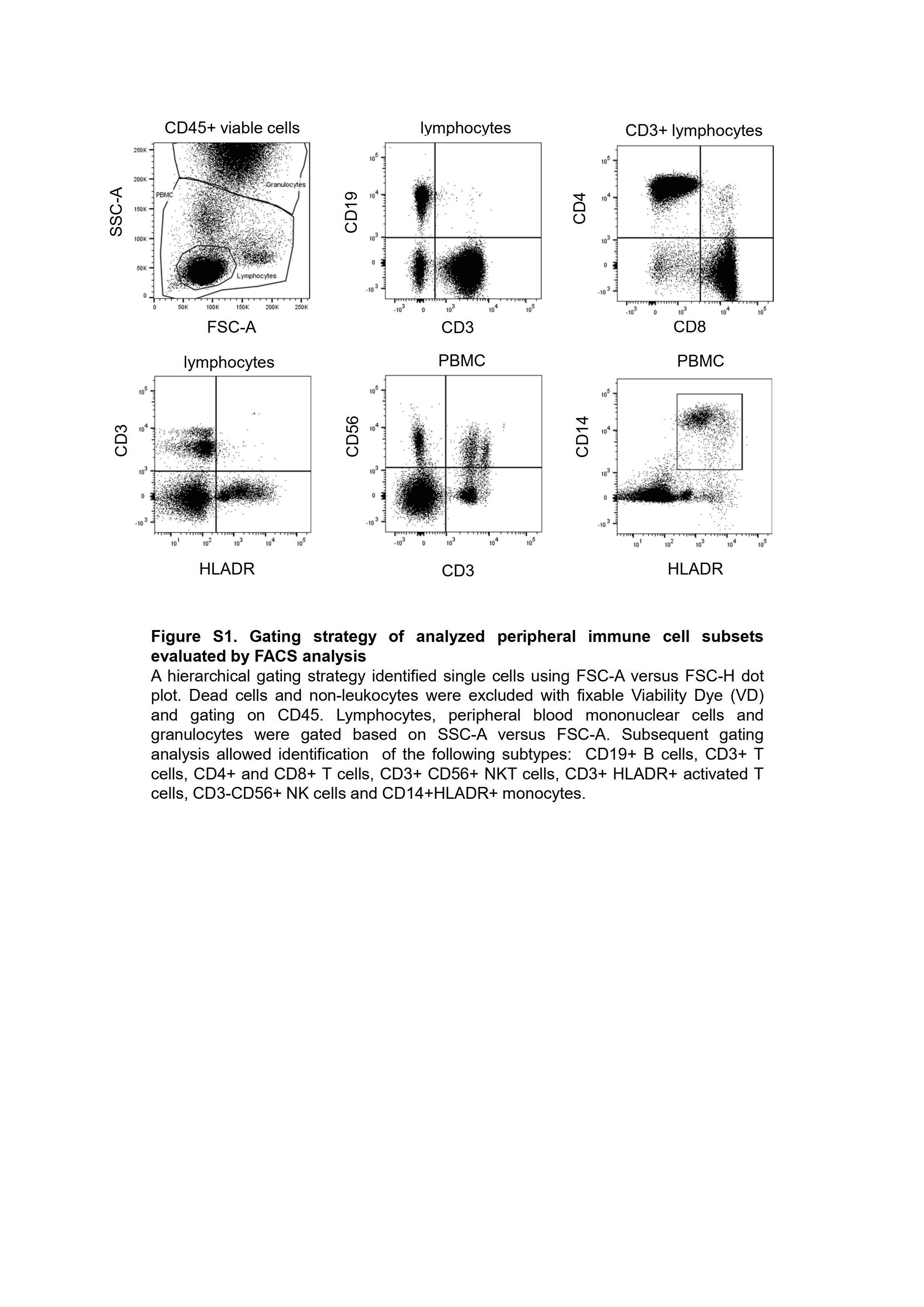

Supplement: Supplementary file 1 [file Image_1.tif]
